# The Small RNA Teg41 Regulates Expression of the Alpha Phenol-Soluble Modulins and Is Required for Virulence in Staphylococcus aureus

**DOI:** 10.1128/mBio.02484-18

**Published:** 2019-02-05

**Authors:** Rachel L. Zapf, Richard E. Wiemels, Rebecca A. Keogh, Donald L. Holzschu, Kayla M. Howell, Emily Trzeciak, Andrew R. Caillet, Kellie A. King, Samantha A. Selhorst, Michael J. Naldrett, Jeffrey L. Bose, Ronan K. Carroll

**Affiliations:** a Department of Biological Sciences, Ohio University, Athens, Ohio, USA; b Proteomics and Metabolomics Facility, Center for Biotechnology, University of Nebraska Lincoln, Lincoln, Nebraska, USA; c Department of Microbiology, Molecular Genetics and Immunology, University of Kansas Medical Center, Kansas City, Kansas, USA; University of Illinois at Chicago

**Keywords:** PSM, *Staphylococcus aureus*, Teg41, phenol-soluble modulin, regulatory RNA, sRNA

## Abstract

Small RNAs (sRNAs) remain an understudied class of regulatory molecules in bacteria in general and in Gram-positive bacteria in particular. In the major human pathogen Staphylococcus aureus, hundreds of sRNAs have been identified; however, only a few have been characterized in detail. In this study, we investigate the role of the sRNA Teg41 in S. aureus virulence. We demonstrate that Teg41, an sRNA divergently transcribed from the locus that encodes the cytolytic alpha phenol-soluble modulin (αPSM) peptides, plays a critical role in αPSM production. Overproduction of Teg41 leads to an increase in αPSM levels and a corresponding increase in hemolytic activity from S. aureus cells and cell-free culture supernatants. To identify regions of Teg41 important for its function, we performed an *in silico* RNA-RNA interaction analysis which predicted an interaction between the 3′ end of Teg41 and the αPSM transcript. Deleting a 24-nucleotide region from the S. aureus genome, corresponding to the 3′ end of Teg41, led to a 10-fold reduction in αPSM-dependent hemolytic activity and attenuation of virulence in a murine abscess model of infection. Restoration of hemolytic activity in the Teg41Δ3′ strain was possible by expressing full-length Teg41 in *trans*. Restoration of hemolytic activity was also possible by expressing the 3′ end of Teg41, suggesting that this region of Teg41 is necessary and sufficient for αPSM-dependent hemolysis. Our results show that Teg41 is positively influencing αPSM production, demonstrating for the first time regulation of the αPSM peptides by an sRNA in S. aureus.

## Abstract (precis)

The alpha phenol-soluble modulins (αPSMs) are among the most potent toxins produced by Staphylococcus aureus. Their biological role during infection has been studied in detail; however, the way they are produced by the bacterial cell is not well understood. In this work, we identify a small RNA molecule called Teg41 that plays an important role in αPSM production by S. aureus. Teg41 positively influences αPSM production. The importance of Teg41 is highlighted by the fact that a strain containing a deletion in the 3′ end of Teg41 produces significantly less αPSMs and is attenuated for virulence in a mouse abscess model of infection. As the search for new therapeutic strategies to combat S. aureus infection proceeds, Teg41 may represent a novel target.

## INTRODUCTION

Staphylococcus aureus is both a commensal of humans and a highly dangerous bacterial pathogen (1). S. aureus pathogenesis is mediated by a large repertoire of secreted and cell wall-associated virulence factors, including a number of potent cytolytic peptides called phenol-soluble modulins (PSMs). PSMs are amphipathic, alpha helical peptides that vary in size depending on their classification. The α type PSMs are typically 20 to 25 amino acids in size, while the β type PSMs are around 40 to 45 amino acids. The α type PSMs have been the focus of intense study in recent years, as they have been implicated in contributing to the high virulence potential of community-acquired methicillin-resistant S. aureus (CA-MRSA) strains (2, 3). While the role of PSMs in S. aureus infection has been extensively investigated (for a comprehensive review of PSMs and S. aureus virulence, see reference 4), their production within the S. aureus cell is less well studied.

**FIG 1:**
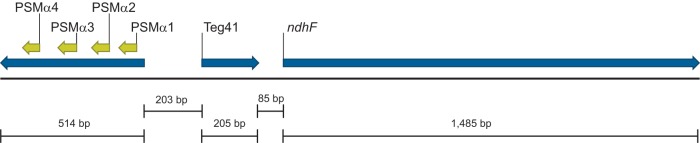
The Teg41 locus in S. aureus. Teg41 is located 203 bp downstream of, and divergently transcribed from, the αPSM operon. The *ndhF* gene is located 85 bp downstream of Teg41 and is transcribed in the same orientation. The Teg41 annotation is based on previously published data (11).

There are five α type PSMs in S. aureus. Four of them (named αPSM1 to αPSM4) are encoded on a polycistronic transcript (the αPSM transcript [Fig. 1]), while the fifth, the δ-toxin, is encoded within the RNAIII transcript. Interestingly, both of these transcripts are regulated by the *agr* system by direct binding of AgrA to the promoter region (5). Therefore, while the production of α type PSMs is linked to population density and virulence (through the *agr* system), little else is known about the mechanisms that control PSM production within the bacterial cell. PSMs do not contain a secretion signal sequence and are secreted from the cell via a dedicated ABC transport system (the Pmt system) (6).

Small RNAs (sRNAs) represent an understudied class of regulatory molecules in S. aureus (7–10). They are often overlooked because the majority of sRNA genes (including that of RNAIII) are not included in GenBank genome annotation files (due to the small size of the αPSM ORFs, the αPSM locus/transcript is typically absent in S. aureus annotated genome files too). To address this, we recently performed a comprehensive mapping study which annotated 303 individual sRNAs (and the αPSM transcript) on the genome of S. aureus CA-MRSA strain USA300 (11). These annotated files serve as a valuable resource for analysis of sRNAs in S. aureus. In addition to providing a reference genome that can be used for transcriptomic studies (of sRNAs and αPSMs), these files clearly outline the location on the chromosome of each sRNA gene relative to protein-coding genes.

During the annotation process, we observed that the gene encoding a previously unstudied sRNA (called Teg41) was located immediately downstream of, and divergently transcribed from, the αPSM transcript (Fig. 1). The close proximity of Teg41 to the αPSMs led us to hypothesize that Teg41 may play a role in regulating αPSM production. In this study, we demonstrate that Teg41 positively regulates expression of the αPSMs. Overproduction of Teg41 leads to increased erythrocyte hemolysis and increased production of αPSMs. We identify the region of Teg41 responsible for regulating the αPSMs and demonstrate that deletion of this region (i) results in a decrease in αPSM production and (ii) attenuates S. aureus virulence in a murine abscess model of infection. Together, these data demonstrate for the first time regulation of the αPSMs by an sRNA and further demonstrate the important role played by sRNAs in regulating virulence in S. aureus.

## RESULTS

### The sRNA Teg41 contributes to hemolytic activity in S. aureus.

**FIG 2:**
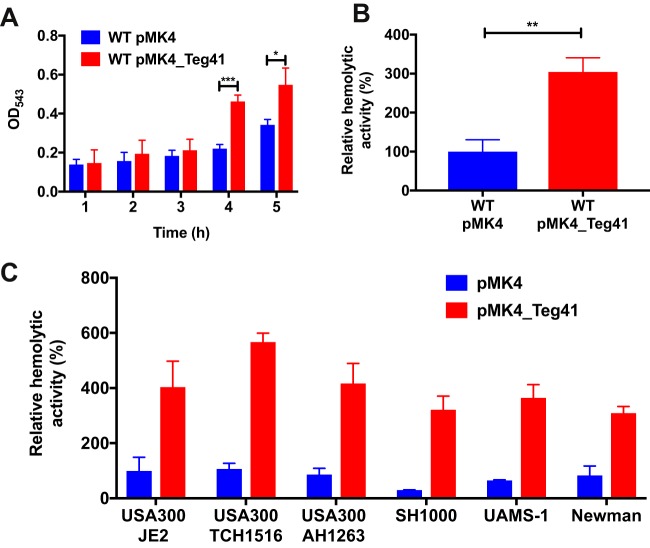
Overproduction of Teg41 leads to increased hemolytic activity in S. aureus. (A) Hemolytic activity of S. aureus cells in human blood is increased upon Teg41 overproduction. Human blood samples were inoculated with S. aureus containing a vector control (wild-type [WT] pMK4) or Teg41-overproducing plasmid (WT pMK4_Teg41). At the time points indicated, the degree of hemolytic activity was determined. No difference in hemolysis was observed at early time points; however, a significant difference was observed at 4 h and 5 h postinoculation. No difference in bacterial numbers was detected at any of the time points. The strain background was TCH1516. (B) Hemolytic activity of cell-free S. aureus 15-h culture supernatants is increased upon Teg41 overproduction. The strain background was TCH1516. (C) Overproduction of Teg41 results in an increase in hemolytic activity in cell-free 15-h culture supernatants in various wild-type S. aureus backgrounds. Hemolytic activity in the wild-type strain containing the vector control (WT pMK4) is set at 100%, and the relative hemolytic activity of the Teg41-overproducing strain (WT pMK4_Teg41) is indicated as a percentage. All statistical analyses were performed using Student’s *t* test. Statistical significance is indicated by bars and asterisks as follows: *, *P* < 0.05; **, *P* < 0.01; ***, *P* < 0.005.

Recently, we performed a comprehensive reannotation of the S. aureus genome in which all previously identified sRNAs were mapped to their reported chromosomal locations (11). During the annotation process we observed that the gene encoding a previously unstudied sRNA (called Teg41) was located immediately downstream of, and divergently transcribed from, the alpha phenol-soluble modulin (αPSM) transcript (Fig. 1). The close proximity of Teg41 to the αPSMs led us to hypothesize that Teg41 may play a role in regulating αPSM production. To test this hypothesis, we attempted to create a Teg41 deletion strain by allelic exchange. Despite repeated attempts, we were unable to delete the Teg41 gene. In the absence of a Teg41 mutant strain, we elected to examine the effect of Teg41 overproduction. The Teg41 gene, under the control of its native promoter, was expressed on a multicopy plasmid (pMK4) in S. aureus strain USA300 TCH1516. The αPSM peptides are potent hemolysins; therefore, to test the potential contribution of Teg41 to αPSM production, we examined the hemolytic activity of S. aureus cells (Fig. 2A) and cell-free culture supernatants (Fig. 2B). When we compared wild-type S. aureus (containing the empty vector) to the Teg41-overproducing strain, a significant increase in hemolytic activity was observed in both S. aureus cells and cell-free culture supernatants taken from the Teg41-overproducing strain (Fig. 2). This result shows that Teg41 contributes to S. aureus hemolytic activity, potentially by regulating αPSM activity.

To investigate whether Teg41 overexpression leads to increased hemolysis in multiple S. aureus backgrounds, the Teg41 overexpression plasmid and empty vector control were transduced into five wild-type S. aureus strains: USA300 JE2, USA300 AH1263, Newman, SH1000, and UAMS-1. Cell-free supernatants from all strains containing the Teg41 overexpression plasmid displayed increased hemolysis compared to vector controls (Fig. 2C), demonstrating that Teg41 overexpression increases hemolysis in multiple S. aureus strains.

**FIG 3:**
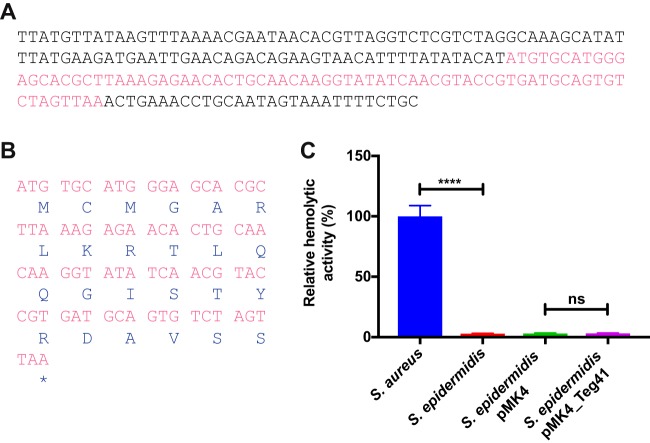
Teg41 does not encode a hemolytic peptide. (A) The Teg41 DNA sequence is shown with the potential ORF highlighted in pink. (B) Translation of Teg41 ORF. A 24-amino-acid peptide is potentially encoded. (C) Expression of Teg41 in S. epidermidis does not result in increased hemolytic activity. Hemolytic activity in S. aureus strain JE2 is shown as a positive control (set at 100%), and the relative hemolytic activity of S. epidermidis strains is indicated.

Overproduction of Teg41 could result in increased hemolytic activity due to a regulatory function, or alternatively Teg41 could itself encode a hemolytic peptide. When we examined the Teg41 sequence, we identified one potential open reading frame (ORF) capable of encoding a peptide of 24 amino acids (Fig. 3A and B). To investigate whether Teg41 elaborates this peptide (and it possesses hemolytic activity), we transduced the Teg41-overproducing plasmid into Staphylococcus epidermidis and performed hemolysis assays using cell-free culture supernatants. No hemolytic activity was observed in S. epidermidis, strongly suggesting that Teg41 does not encode a hemolytic peptide (Fig. 3C). Taken together, the data above indicate that overexpression of Teg41 leads to an increase in hemolytic activity in staphylococcal strains carrying genes that encode the αPSM peptides.

### Teg41 is highly conserved in S. aureus.

The Teg41 transcript was first identified by Beaume et al. in 2010 (12). Prior to that, work by Geissman et al. had predicted the existence of an sRNA at the corresponding location (which they termed RsaX05), due to the presence of orphan promoter and terminator sequences (13). Teg41 transcript has subsequently been identified in a number of published RNAseq experiments and has been given a variety of different names and designations (including sRNA095 [14], srn_1080 [15], and SAUSA300s087 [11]). Here we refer to it as Teg41, the name used upon the first experimental identification of the sRNA (12). While Teg41 has been observed in a number of studies, there are no reports investigating the role of Teg41 in S. aureus.

Teg41 was reported by Geissman et al. (13) and Beaume et al. (12) as a transcript located between the genes for the αPSMs and *ndhF*. *ndhF* encodes an NADH dehydrogenase involved in the transfer of electrons from NAD to quinones in the cellular membrane (16). Due to its close proximity and similar orientation to *ndhF*, it was speculated that Teg41 may function as a *cis*-acting riboswitch or 5′ UTR for this gene (Fig. 1) (12, 13). The results presented herein (Fig. 2A and below) suggest that this is not the case, and instead, they imply that Teg41 is a stand-alone transcript that influences hemolytic activity of S. aureus.

The sequence of Teg41 is highly conserved in S. aureus. A BLAST search of the Teg41 sequence from S. aureus strain USA300 FPR3757 identified 161 Teg41 homologues that displayed 100% sequence identity. The minimum sequence identity of S. aureus Teg41 homologues in the database is 94%. Outside of S. aureus, Teg41 is found in only one other *Staphylococcus* species, the closely related Staphylococcus argenteus. Interestingly, this pattern of distribution among staphylococcal strains (i.e., present only in S. aureus and S. argenteus) mirrors that of the αPSM operon. In contrast to this, *ndhF*, the gene downstream of Teg41, is found in all staphylococcal species. Thus, the αPSM operon and Teg41 are genetically linked, while a number of staphylococcal spp. contain a copy of the *ndhF* gene that is not preceded by Teg41.

### Overproduction of Teg41 in a *psmα* mutant does not result in increased hemolytic activity.

**FIG 4:**
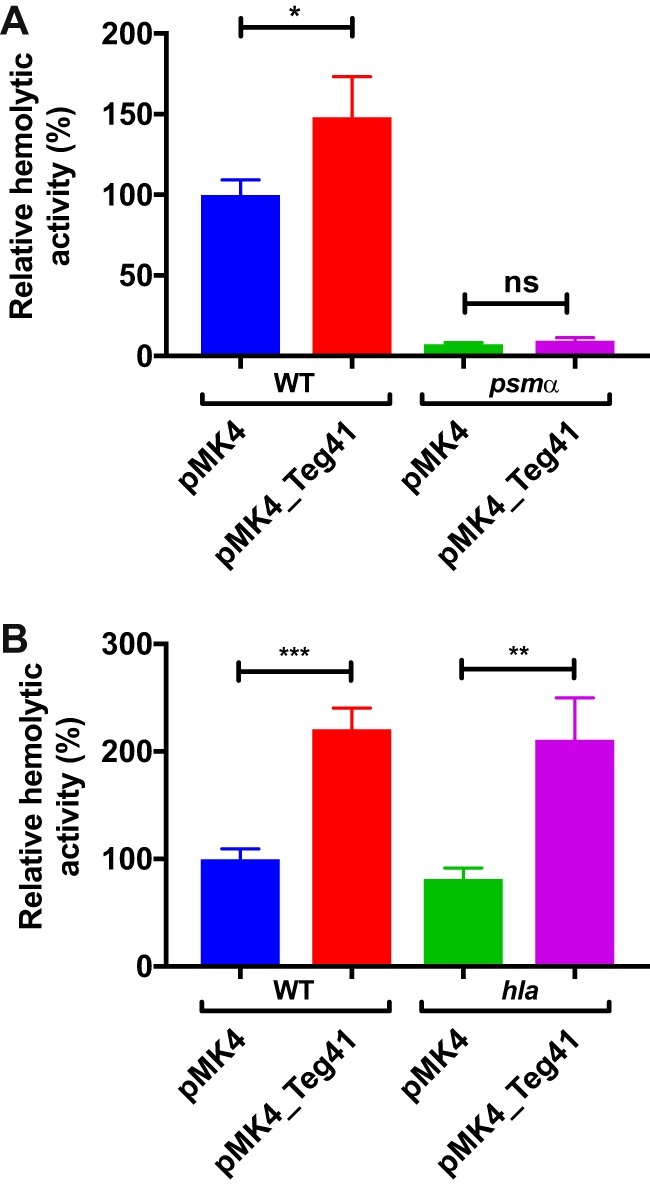
Teg41 overproduction leads to an increase in αPSM-dependent hemolysis. (A) Overproduction of Teg41 leads to a significant increase in hemolysis in the wild-type background (WT pMK4 compared to WT pMK4_Teg41); however, overproduction of Teg41 in an αPSM mutant (*psmα* pMK4_Teg41) does not result in increased hemolytic activity compared to the αPSM mutant with the vector control (*psmα* pMK4). The strain background was JE2. (B) Overproduction of Teg41 leads to a significant increase in hemolysis in the wild-type background (WT pMK4 compared to WT pMK4_Teg41). A similar increase in hemolytic activity is observed upon overproduction of Teg41 in an *hla* mutant (*hla* pMK4 compared to *hla* pMK4_Teg41). The strain background was AH1263. Hemolytic activity in the wild-type strain containing the vector control (WT pMK4) is set at 100%, and the relative hemolytic activity of all other strains is indicated as a percentage. Statistical analyses were performed using Student’s *t* test. Statistical significance is indicated as follows: *, *P* < 0.05; **, *P* < 0.01; ***, *P* < 0.005; ns, not significant.

Due to the increase in hemolytic activity observed upon Teg41 overproduction and the close proximity of Teg41 to the *psmα* gene, we hypothesized that Teg41 is regulating αPSM production. To test this hypothesis, we overexpressed Teg41 in a *psmα* mutant strain (i.e., one in which the entire *psmα* locus has been deleted). If the hemolytic phenotype observed upon Teg41 overproduction is independent of the αPSMs, then an increase in hemolytic activity would be expected upon Teg41 overproduction in a *psmα* mutant strain. Results demonstrated that this was not the case. Overproduction of Teg41 in the JE2 wild-type S. aureus strain resulted in an increase in hemolysis, similar to that observed previously (Fig. 2); however, overproduction of Teg41 in the *psmα* mutant strain did not result in a significant increase in hemolytic activity (Fig. 4A). This result shows that the increase in hemolysis observed following overproduction of Teg41 is dependent on the αPSMs. Furthermore, it confirms that the increase in hemolysis is not due to a Teg41 encoded peptide, as no increase in hemolytic activity was observed in the *psmα* mutant strain overproducing Teg41.

To confirm that Teg41 overproduction leads to increased αPSM-dependent hemolysis, we overproduced Teg41 in a *hla* mutant strain. The *hla* gene encodes the S. aureus α-hemolysin (Hla), another potent hemolytic toxin. Overproduction of Teg41 in the *hla* mutant led to an increase in hemolytic activity, similar to that observed in the wild-type strain (Fig. 4B), clearly demonstrating that the increase in hemolysis observed is not due to the activity of Hla. It should be noted that *hla* mutant strains are still hemolytic (Fig. 4B). This is consistent with studies that demonstrate that Hla is highly hemolytic toward rabbit and sheep erythrocytes but relatively inactive against human erythrocytes (17).

### Overproduction of Teg41 results in increased αPSM production.

**FIG 5:**
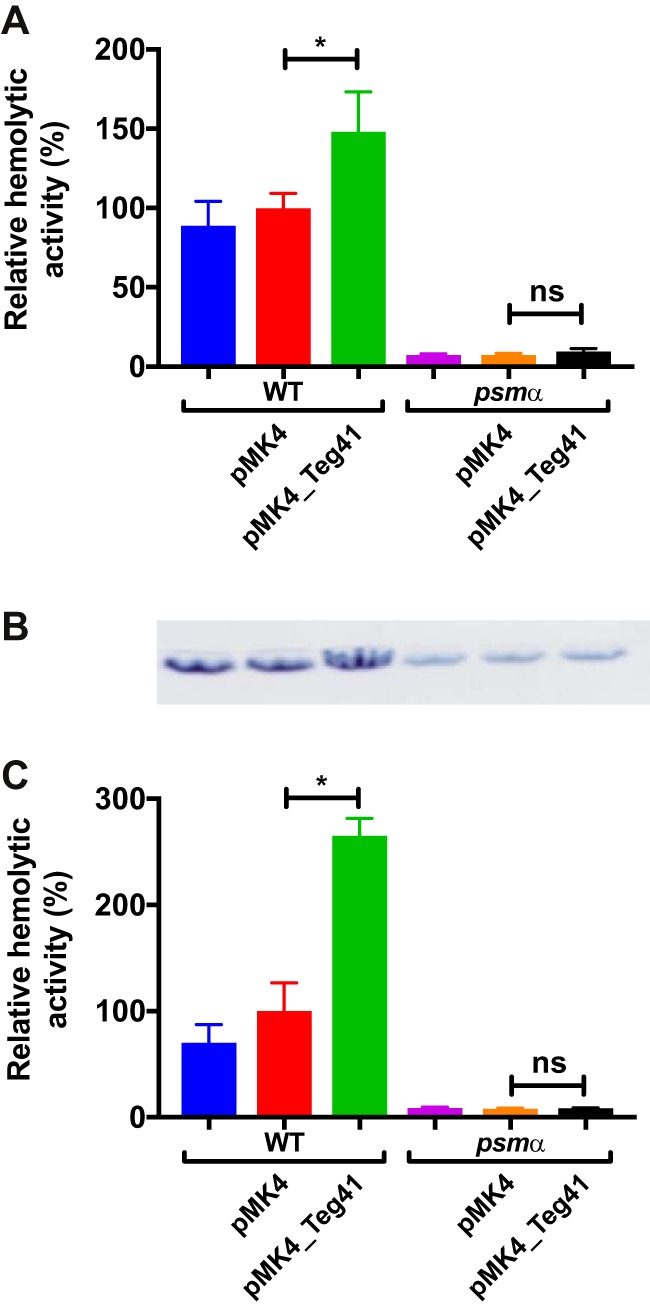
Overproduction of Teg41 leads to increased αPSM levels. (A) Hemolytic activity of S. aureus culture supernatants prior to butanol extraction. No difference in hemolytic activity was observed for strains with and without the vector plasmid. Overproduction of Teg41 leads to a significant increase in hemolysis in the wild-type background (WT pMK4 compared to WT pMK4_Teg41) but not in the αPSM mutant (*psmα* pMK4 compared to *psmα* pMK4_Teg41). (B) Coomassie blue-stained SDS-PAGE analysis of butanol extracts. A band corresponding in size to the PSMs was observed in all strains. Bands of similar intensities were observed in the wild-type strain with and without the empty vector. A band with increased intensity was observed in the wild-type strain overproducing Teg41. An overall decrease in PSM levels was observed in the αPSM mutant, which did not vary upon overproduction of Teg41. (C) Hemolytic activity of butanol-extracted peptides. Butanol-extracted samples were dissolved in water and used in human blood hemolysis assays. A similar result was obtained to that shown in panel A (using culture supernatants), indicating that the hemolysin responsible was purified during the butanol extraction process. Hemolytic activity in the wild-type strain containing the vector control (WT pMK4) is set at 100%, and the relative hemolytic activity of all other strains is indicated as a percentage. Statistical analyses were performed using Student’s *t* test. Statistical significance is indicated as follows: *, *P* < 0.05; ns, not significant. The strain background was JE2.

The results above strongly suggest that increased Teg41 production leads to increased αPSM levels. To test this hypothesis and determine whether αPSM peptide levels are increased upon Teg41 overproduction, we performed a butanol extraction to isolate and purify the PSM peptides from S. aureus culture supernatants (18). The extracted peptides were used in hemolysis assays and analyzed by SDS-PAGE (Fig. 5). The relative degree of hemolytic activity of extracted peptides (Fig. 5C) was similar to that observed in cell-free culture supernatants before butanol extraction (Fig. 5A). These results indicate that the Teg41-dependent hemolysin was isolated during the butanol extraction procedure.

SDS-PAGE analysis of butanol extractions shows one band corresponding to the PSMs (Fig. 5B; see also Fig. S1 in the supplemental material). An increase in PSM abundance was observed when Teg41 was overproduced in the wild-type background (Fig. 5B). Densitometry analysis on serially diluted samples indicates that PSM levels are increased approximately1.8-fold when Teg41 was overproduced (Fig. S2). An overall decrease in PSM levels was observed in the *psmα* mutant strain; however, a band was still detected (presumably representing the δ-toxin). Overproduction of Teg41 in the *psmα* mutant strain did not result in increased PSM production.

10.1128/mBio.02484-18.1 FIG S1 Full-length gel from Fig. 5B. Download FIG S1, PDF file, 0.9 MB. Copyright © 2019 Zapf et al. 2019 Zapf et al. This content is distributed under the terms of the Creative Commons Attribution 4.0 International license.

10.1128/mBio.02484-18.2 FIG S2 Densitometry analysis of butanol-extracted PSMs. Download FIG S2, PDF file, 2.0 MB. Copyright © 2019 Zapf et al. 2019 Zapf et al. This content is distributed under the terms of the Creative Commons Attribution 4.0 International license.

To investigate further the nature and composition of the peptides visualized by SDS-PAGE, butanol-extracted peptides from the wild-type and *psmα* mutant strains with and without Teg41 overproduction were subjected to mass spectrometry analysis. Fragment ion spectra corresponding to the delta-toxin (Hld) peptide were identified in all samples, confirming that the band visible in the *psmα* mutant strains is Hld. In addition to Hld, peptides corresponding to αPSM1 to -4 were detected in extracts from wild-type bacteria, but not from the *psmα* mutant. This confirms that Teg41 overproduction leads to an increase in αPSM production, which in turn accounts for the increase observed in hemolysis.

### The 3′ region of Teg41 is required for hemolytic activity.

**FIG 6:**
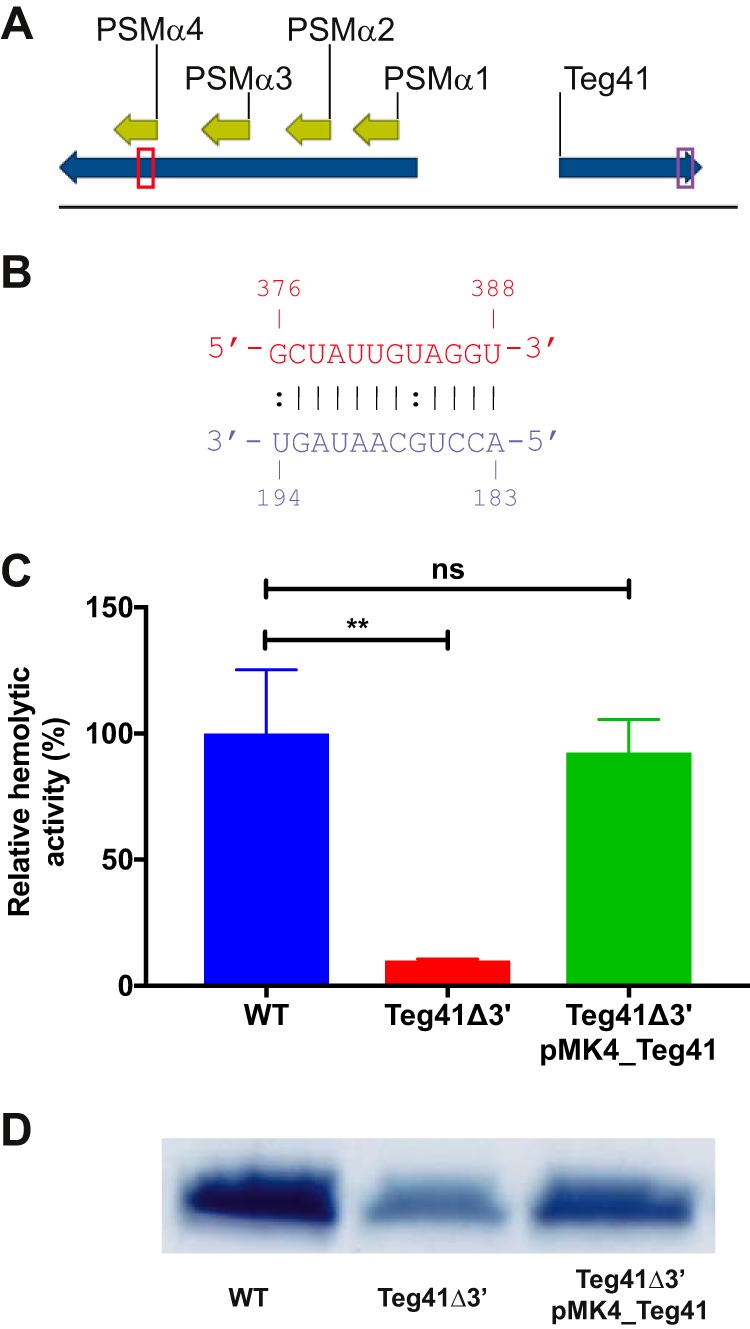
The 3′ end of Teg41 is required for hemolytic activity. (A and B) *In silico* analysis predicts an interaction between the 3′ end of Teg41 (nucleotides 183 to 194; colored purple) and a region of the αPSM transcript downstream of the PSMα4 translation start site (nucleotides 376 to 388; colored red). (A) The αPSM-Teg41 locus. The regions of each transcript predicted to interact are boxed. (B) Predicted interaction between the αPSM transcript (red) and Teg41 (purple). Canonical RNA-RNA base pairs are indicated by vertical lines; noncanonical base pairing is indicated by colons. (C) Hemolytic activity of S. aureus culture supernatants is significantly reduced in the Teg41Δ3′ strain. A 10-fold reduction in hemolytic activity was observed in culture supernatants from the Teg41Δ3′ strain. Hemolysis was restored to wild-type levels by introducing full-length Teg41 on a plasmid (Teg41Δ3′ pMK4_Teg41). Hemolytic activity in the wild-type strain is set at 100%, and the relative hemolytic activity of the other strains is indicated as a percentage. Statistical analyses were performed using Student’s *t* test (**, *P* < 0.01; ns, not significant). (D) PSM levels are significantly reduced in the Teg41Δ3′ strain. Culture supernatants from panel C were butanol extracted and analyzed by SDS-PAGE. A reduction in PSM peptide levels was observed in the Teg41Δ3′ strain. Expressing full-length Teg41 from a plasmid in the Teg41Δ3′ strain (Teg41Δ3′ pMK4_Teg41) resulted in an increase in PSM production. The strain background was AH1263.

To investigate the molecular mechanism through which Teg41 regulates the αPSMs, we began by analyzing the sequences of Teg41 and the αPSM transcript. We used the program intaRNA (19–21) to search for potential interactions between these two RNA molecules. The results generated predict an interaction between the 3′ region of Teg41 (nucleotides 183 to 194) and the αPSM transcript between nucleotides 376 and 388 (Fig. 6A and B). Interestingly, this region of the αPSM transcript is located immediately downstream of the translation start codon for PSMα4 (Fig. 6B). On the basis of the results presented so far and the predicted interaction between Teg41 and the αPSM transcript, we hypothesized that a direct interaction between the 3′ region of Teg41 and the αPSM transcript increases αPSM peptide production. To test this hypothesis, we constructed a mutant strain in which 24 nucleotides in the 3′ region of Teg41 were deleted (i.e., the region predicted to interact with the αPSM transcript). As previously mentioned, our attempts to create a complete Teg41 deletion strain were unsuccessful; however, our attempt to delete the 3′ region of Teg41 (Teg41Δ3) was successful.

To test the hypothesis that deletion of the αPSM interaction site of Teg41 would lead to a decrease in hemolysis, we examined the hemolytic activity of culture supernatants from the Teg41Δ3′ strain. Results show a 10-fold reduction in hemolytic activity in the Teg41Δ3′ strain compared to the wild type (Fig. 6C). Providing full-length Teg41 in *trans*, on a plasmid, completely restored hemolytic activity in the Teg41Δ3′ strain. To confirm that the results of the hemolysis assay in Fig. 6C were due to alterations in PSM production, culture supernatants were subjected to butanol extraction. SDS-PAGE analysis of the extracted peptides shows a reduction in PSM levels in the Teg41Δ3′ strain (Fig. 6D). Providing Teg41 in *trans* (Teg41Δ3′ pMK4_Teg41) increased PSM levels although not completely to the level in the wild-type strain. Together, these results clearly demonstrate that Teg41 is required for S. aureus αPSM production and hemolytic activity. Moreover, the results are consistent with a model whereby the 3′ end of Teg41 is responsible for modulating αPSM levels.

### Teg41 is required for virulence in an abscess model of infection.

**FIG 7:**
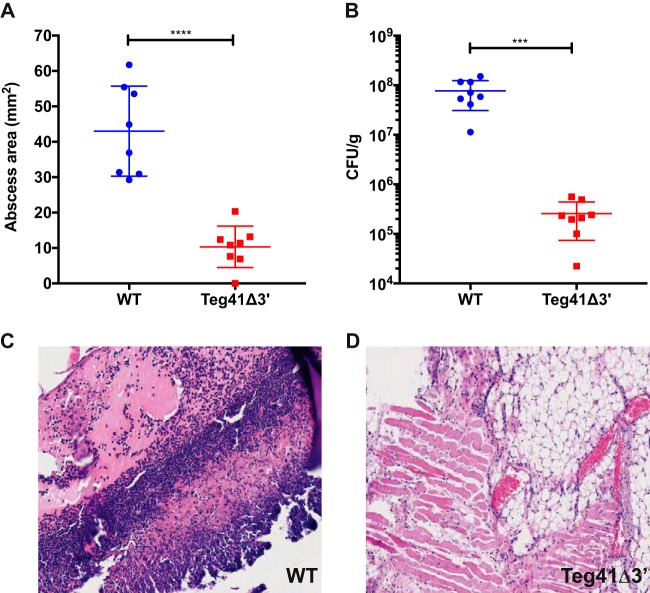
Teg41 contributes to virulence in S. aureus. Wild-type S. aureus and the Teg41Δ3′ strain were inoculated subcutaneously into groups of 12 mice. (A and B) Abscess size (A) and the number of bacteria present in abscesses (CFU/gram) (B) was determined following a 7-day infection period. A 4.2-fold reduction in abscess size and a 302-fold reduction in bacterial numbers was observed in the abscesses of mice infected with Teg41Δ3′ compared to those infected with the wild-type strain. (C) After histopathological staining, the abscesses of mice infected with the wild-type strain displayed a large amount of infiltrating inflammation-associated leukocytes (stained purple) and necrotic muscle tissue (stained pink). (D) In contrast, mice infected with Teg41Δ3′ displayed abscesses with a low population of leukocytes and intact muscle tissue with no necrotic areas. All pictures were taken at a magnification of ×20. Statistical significance was determined using Student’s *t* test (***, *P* < 0.005; ****, *P* < 0.001). The strain background was AH1263.

The results presented above (Fig. 6C and D) demonstrate a reduction in PSM production and hemolytic activity when Teg41 is disrupted. To compare the virulence of wild-type S. aureus and the Teg41Δ3′ strain, we employed a murine abscess model of infection. Groups of 12 mice were subcutaneously injected with S. aureus, and following a 7-day infection, abscesses were measured and the number of bacteria present was determined. Abscess area was significantly reduced in mice infected with the Teg41Δ3′strain compared to the wild type (Fig. 7A). Additionally, a 302-fold reduction in bacterial numbers was observed in the abscesses of mice infected with the Teg41Δ3′ strain compared to those infected with the wild type (Fig. 7B). Histopathology of excised abscesses shows widespread necrosis and muscle destruction in mice infected with the wild-type strain, as well as a high concentration of inflammation-associated leukocytes (Fig. 7C). Conversely, mice infected with the Teg41Δ3′strain display intact muscle tissue, no necrosis, and a lower concentration of leukocytes (Fig. 7D). Taken together, these results show that the reduction in αPSM production in the Teg41Δ3′ strain results in attenuation and clearly demonstrates that Teg41 is required for virulence in S. aureus. Growth curve analysis demonstrated that that there is no difference in growth rate between the wild-type and Teg41Δ3′ strains (Fig. S3).

10.1128/mBio.02484-18.3 FIG S3 Growth of wild-type S. aureus and the Teg41Δ3′ mutant in TSB. Download FIG S3, PDF file, 0.8 MB. Copyright © 2019 Zapf et al. 2019 Zapf et al. This content is distributed under the terms of the Creative Commons Attribution 4.0 International license.

**FIG 8:**
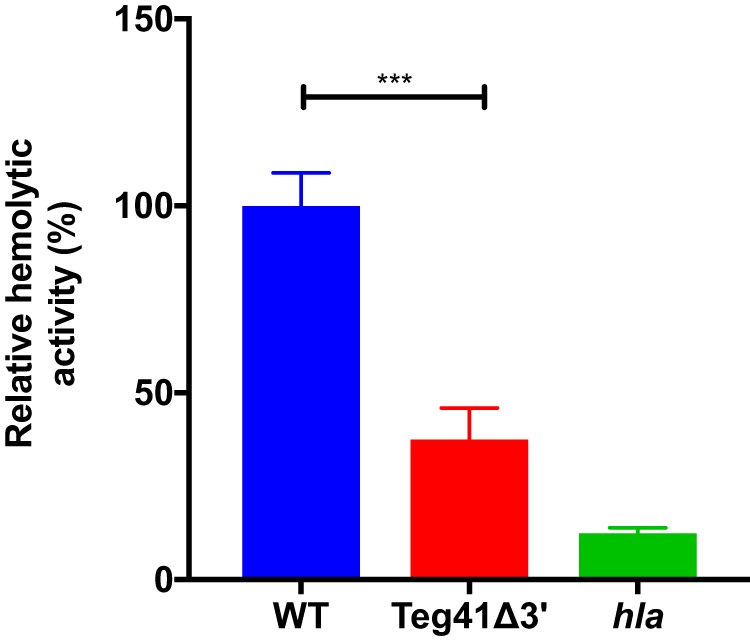
The Teg41Δ3′ strain demonstrates decreased rabbit erythrocyte lysis. Compared to the wild type, Teg41Δ3′ displays decreased lysis of rabbit erythrocytes after a 5-min incubation at 37°C. Although significant, the reduction in hemolytic activity was not as pronounced as in an *hla* mutant strain. Hemolytic activity in the wild-type strain (WT) is set at 100%, and the relative hemolytic activity of all other strains is indicated as a percentage. Statistical analyses were performed using Student’s *t* test. Statistical significance is indicated as follows: ***, *P* < 0.005. The strain background was AH1263.

Virulence of S. aureus in the murine abscess model is strongly linked to the activity of the alpha-toxin, Hla. Hla production has been shown to be dependent on αPSM levels, with delayed induction of *hla* expression observed in an αPSM mutant strain (22). Therefore, we hypothesized that the attenuation in virulence observed in the Teg41Δ3′ strain may be due in part to decreased Hla production. To test this hypothesis, we performed hemolysis assays using S. aureus culture supernatants and rabbit blood (as previously mentioned, rabbit erythrocytes are exquisitely sensitive to Hla-mediated lysis). Results show a reduction in activity from Teg41Δ3′ culture supernatants compared to the wild-type culture supernatant, strongly suggesting reduced Hla production in this strain (Fig. 8).

To confirm that the virulence phenotypes observed in the Teg41Δ3′ strain were due to the 24-nt deletion in Teg41, we subjected the Teg41Δ3′ strain and the parental wild-type strain to whole-genome sequencing. Analysis of the genome sequencing data confirmed the deletion in the 3′ region of Teg41. No additional polymorphisms, insertions, or deletions were observed in any S. aureus virulence genes and/or regulatory proteins in the Teg41Δ3′ strain.

### The 3′ end of Teg41 is necessary and sufficient for S. aureus hemolysis.

**FIG 9:**
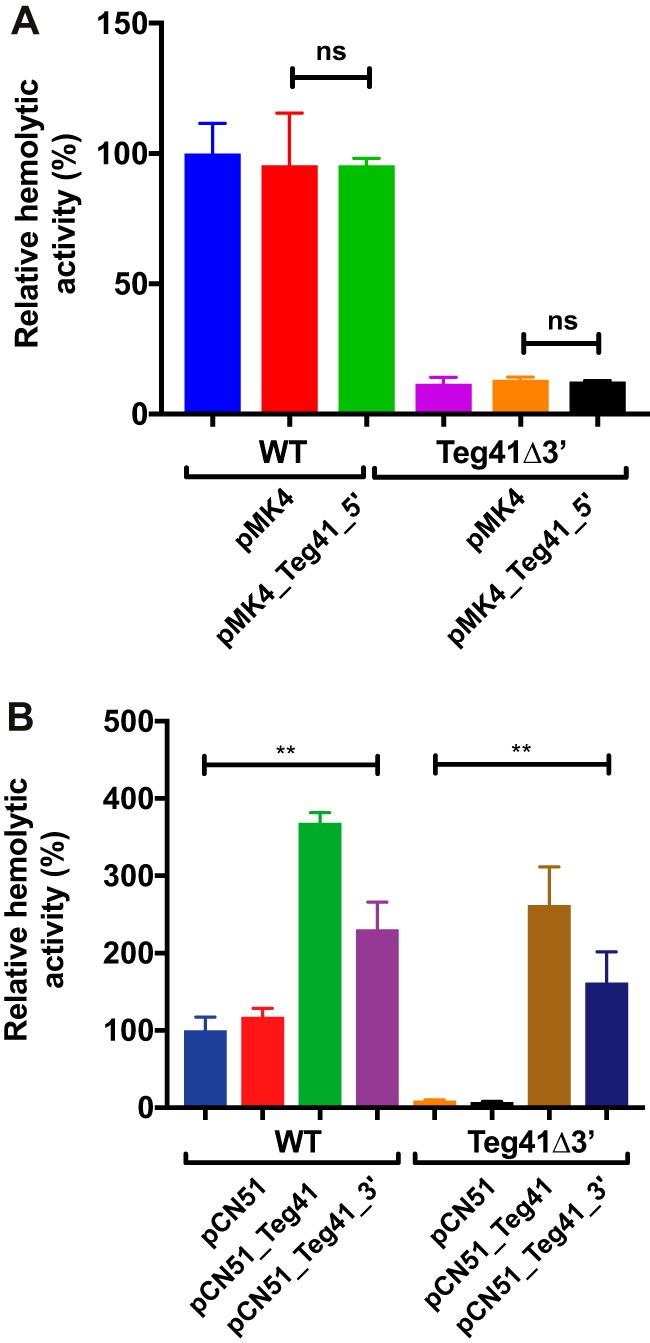
The 3′ end of Teg41 is necessary and sufficient for S. aureus hemolytic activity. (A) Expression of truncated Teg41 (pMK4_Teg41_5) does not result in an increase in hemolytic activity in either the wild-type or Teg41Δ3′ strain compared to empty vector (pMK4) controls or strains without plasmids. (B) Overexpression of full-length Teg41 (pCN51_Teg41) or the Teg41 3′ end (pCN51_Teg41_3’) results in an increase in hemolytic activity in both wild-type S. aureus and the Teg41Δ3′ strain. In panels A and B, hemolytic activity in the wild-type strain without plasmid was set at 100%, and the relative hemolytic activity of all other strains was indicated as a percentage. Statistical analyses were performed using Student’s *t* test. Statistical significance is indicated as follows: **, *P* < 0.01; ns = not significant. The strain background was AH1263.

The data presented above (Fig. 6) show that the 3′ end of Teg41 is necessary for αPSM-dependent hemolytic activity in S. aureus. Expression of full-length Teg41 from a plasmid restored hemolysis and PSM production in the Teg41Δ3′ strain (Fig. 6). To investigate further the role of the 3′ end of Teg41, we constructed a plasmid expressing a truncated form of Teg41 missing 47 nt at the 3′ end (Teg41_5′). When the truncated form of Teg41 (Teg41_5′) was expressed in wild-type S. aureus or in the Teg41Δ3′ strain, there was no increase in hemolytic activity (Fig. 9A). This result specifically shows that the 3′ end of Teg41 is necessary to restore hemolysis when ectopically expressed from a plasmid.

We next wanted to investigate whether expression of the 3′ end of Teg41 alone is sufficient for hemolytic activity. To do so, we examined the ability of the isolated 3′ end of Teg41 (Teg41_3′) to induce hemolysis in the wild-type and Teg41Δ3′ strains. Full-length Teg41 and Teg41_3′ were cloned into the inducible plasmid pCN51 (generating plasmids pCN51_Teg41 and pCN51_Teg41_3′), and each plasmid, along with the vector control, was transformed into wild-type S. aureus and the Teg41Δ3′ strain. Expression from each plasmid was induced by the addition of CdCl_2_, and hemolytic activity of culture supernatants was determined using human blood. As previously demonstrated, overexpression of full-length Teg41 resulted in an increase in hemolytic activity in wild-type S. aureus and restored hemolytic activity in the Teg41Δ3′ strain (Fig. 9B). Interestingly, expression of Teg41_3′ also resulted in an in increase in hemolytic activity and restoration of hemolytic activity in the wild-type and Teg41Δ3′ strains, respectively. This result clearly demonstrates that the 3′ end of Teg41 is both necessary and sufficient for αPSM-mediated hemolytic activity in S. aureus. In both the wild-type and Teg41Δ3′ strains, the increase in hemolytic activity observed from expression of Teg41_3′ was not as great as that observed when expressing full-length Teg41. This suggests that while the hemolysis effect is mediated by the 3′ end of Teg41, the 5′ end may still play a role in Teg41 activity.

### Analysis of Teg41 and αPSM transcripts.

**FIG 10:**
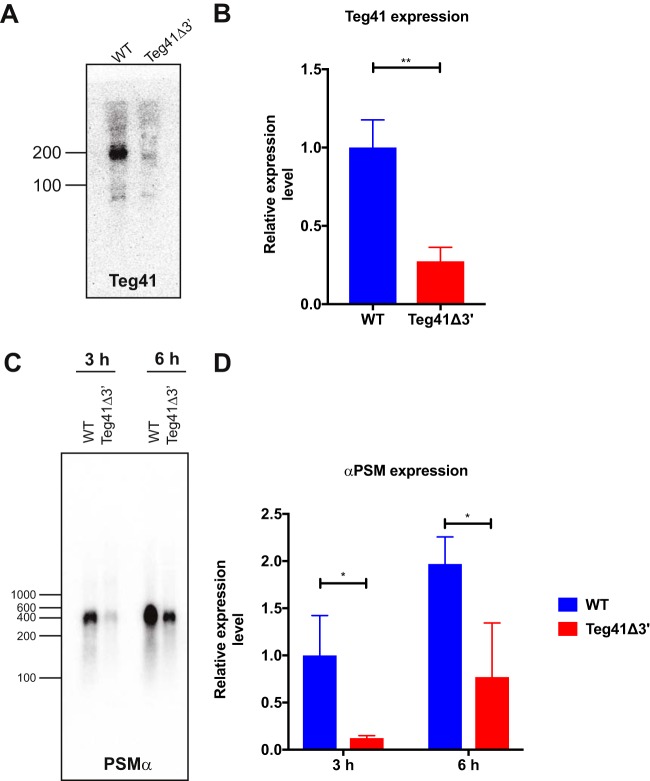
Analysis of Teg41 and αPSM transcripts. (A) Northern blot of Teg41 levels in wild-type S. aureus and the Teg41Δ3′ strain. RNA was extracted from cells growing for 6 h in TSB and probed with a radiolabeled Teg41 probe. (B) RT-qPCR analysis of the 5′ end of Teg41. RT-qPCR was performed using primers that anneal to the 5′ end of Teg41. Data shown are the averages of two technical replicates from three biological replicates. Expression levels are normalized using *gyrB*, and the wild-type sample is set at 1. Error bars represent standard deviations. Statistical analyses were performed using Student’s *t* test. Statistical significance is indicated as follows: **, *P* < 0.01. (C) Northern blot analysis of αPSM levels in wild-type S. aureus and the Teg41Δ3′ strain. RNA was extracted from cells growing for 3 h and 6 h in TSB and probed with a radiolabeled αPSM probe. (D) RT-qPCR analysis of αPSM transcript levels. RT-qPCR was performed on RNA samples identical to those used in panel C (i.e., wild-type S. aureus and the Teg41Δ3′ strain at 3 h and 6 h). Data shown are the averages of two technical replicates from three biological replicates. Expression levels are normalized using *gyrB*, and the wild-type sample is set at 1. Error bars represent standard deviations. Statistical analyses were performed using Student’s *t* test. Statistical significance is indicated as follows: *, *P* < 0.05. The strain background was AH1263.

Teg41 was initially reported, and is annotated, as a 205-nt transcript (12); however, previous studies have reported different sizes, with estimates ranging from 146 nt (14) to 300 nt (13). To confirm the size of Teg41 and investigate the effect of the 24-nt chromosomal deletion in the Teg41Δ3′ strain, we performed Northern blotting using a Teg41-specific probe and total RNA extracted from wild-type S. aureus and the Teg41Δ3′ strain at 6 h growth. Results show a band approximately 200 nt in size in the wild-type bacteria (Fig. 10A), confirming the predicted size of Teg41 (12) and demonstrating that the Teg41 transcript is independent of *ndhF*. In contrast, Teg41 was barely detected in the Teg41Δ3′ strain. A very faint band was detected, running at a slightly lower apparent molecular weight, which we hypothesize is the truncated form of Teg41 (i.e., Teg41_3′). The large reduction in Teg41 abundance in the Teg41Δ3′ strain suggests that truncated Teg41 is probably unstable and is rapidly degraded in the cell. To confirm that the band detected corresponds to truncated Teg41, we performed reverse transcriptase quantitative PCR (RT-qPCR) using primers that anneal to the 5′ end of Teg41 (which is present in the Teg41Δ3′ strain). Using RNA from 6-h growth as the template, results show that truncated Teg41 was detected but that the transcript is approximately threefold less abundant (Fig. 10B). These results show that truncated Teg41 is being expressed in the Teg41Δ3′ strain but is present at a lower level than full-length Teg41 in the wild-type strain.

Our data clearly show that Teg41 positively influences αPSM production. Positive gene regulation by sRNAs is less common than negative regulation and whenever reported often occurs through the stabilization of transcripts, which leads to increased translation. To determine whether the presence of Teg41 leads to increased abundance of the αPSM transcript, we performed Northern blotting to examine αPSM transcript levels in the wild-type and Teg41Δ3′ strain. Using RNA extracted at 3 h and 6 h of growth, we observed decreased αPSM transcript levels in the Teg41Δ3′ strain compared to the wild type (Fig. 10C). The reduction in αPSM transcript levels in the Teg41Δ3′ strain was confirmed by RT-qPCR (Fig. 10D).

Together, these results show that deleting the 3′ end of Teg41 leads to decreased Teg41 and αPSM transcript levels in the cell. While the decrease in αPSM transcript levels could be due to decreased promoter activity, we hypothesize that in the absence of Teg41, the αPSM transcript is rapidly degraded, leading to decreased αPSM peptide production and attenuation of virulence.

## DISCUSSION

**FIG 11:**
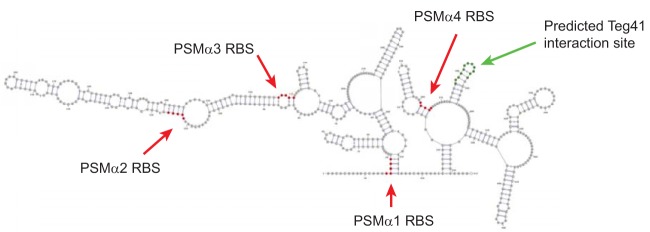
Predicted secondary structure of the αPSM transcript. The locations of each αPSM RBS are indicated in red. The region predicted to interact with Teg41 is shown in green.

Over the last decade, the biological role and contribution of the αPSMs to S. aureus virulence have become clear, but there are still significant gaps in our understanding of how they are produced in the bacterial cell. Although PSMα1 to PSMα4 are encoded within the same polycistronic transcript (Fig. 1), recent *in vitro* studies have shown that the levels of the four peptides vary. A study of MRSA isolates demonstrated that PSMα4 is commonly the most abundant αPSM, followed by PSMα1 (23). PSMα2 and PSMα3 are typically the least abundant. A similar pattern was reported in MSSA strains (24). The mechanism responsible for this variation in αPSM levels is unknown; however, this study and the discovery that the αPSM transcript is subject to sRNA-mediated regulation may begin to shed some light on it. For the first time (that we are aware of), we have demonstrated sRNA-mediated regulation of the αPSM peptides. Teg41 positively influences αPSM production with αPSM levels increased upon Teg41 overproduction and decreased in the Teg41Δ3′ strain (i.e., when the 3′ region of Teg41 is deleted). Our Northern blot analysis shows decreased abundance of the αPSM transcript in the Teg41Δ3′ strain, suggesting that Teg41 regulates the αPSMs at the level of transcription or transcript stability. sRNA-mediated, positive gene regulation frequently occurs at the level of transcript stability, leading us to propose a model whereby binding of Teg41 to the αPSM transcript stabilizes the transcript, facilitating increased translation of the αPSM peptides. This proposed model does not preclude the possibility that Teg41 also functions at the translational level. Binding of Teg41 could result in a conformational change to the αPSM transcript that increases or decreases ribosomal access to one or more αPSM ribosome binding sites. In support of this idea, the predicted structure of the αPSM transcript shows all four αPSM ribosome-binding sites (RBS) located within base-paired regions (Fig. 11). Thus, while a transcript stability mechanism seems likely, further experimental investigation is necessary to fully understand the mechanism(s) underlying Teg41-mediated regulation of the αPSMs. Interestingly, a recent bioinformatic analysis of published RNAseq data, performed by Subramanian et al. (25), examined the expression profile of sRNAs in S. aureus and predicted an important role for Teg41 (called sRNA095 in their study) in S. aureus pathogenesis. Here we provide experimental evidence to confirm this prediction, validating the approach used in this study.

The sequences of sRNAs typically give little valuable information regarding their nature or function. Initial reports suggested that Teg41 was likely a 5′ UTR or riboswitch for the downstream *ndhF* gene (12, 13). Our analysis also revealed the possibility that a short peptide was encoded within the Teg41 RNA. The data presented in this study clearly demonstrate that neither of these is the case. Rather, Teg41 is a stand-alone, *trans*-acting regulatory RNA that regulates production of the αPSMs. While it is impossible to rule out the existence of a hemolytic Teg41-derived peptide, three important results demonstrate that this potential peptide is not contributing to hemolysis. First, overproduction of Teg41 in Staphylococcus epidermidis did not result in an increase in hemolytic activity (Fig. 3). Second, overproduction of Teg41 in an αPSM mutant did not result in an increase in hemolytic activity (Fig. 4). If Teg41 were to encode a hemolytic peptide, it is highly likely that an increase in hemolysis would have been observed in these experiments. Finally, expressing the 3′ end of Teg41 from a plasmid (pCN51_Teg41_3’) caused an increase in hemolytic activity in the wild-type and Teg41Δ3′ strain. The Teg41_3′ segment expressed from this plasmid does not contain the potential ORF, thereby conclusively demonstrating that a hemolytic peptide is not responsible for the Teg41-mediated increase in hemolysis observed.

The αPSM operon is a rare example of a locus in S. aureus that is regulated by the *agr* system independently of RNAIII (5). The response regulator AgrA binds directly to the αPSM promoter and activates transcription. This ties αPSM expression to cell density. A second virulence-associated global regulator, MgrA, was recently shown to bind to the αPSM promoter and regulate expression of the αPSM operon (26). In contrast to AgrA, MgrA represses expression of the αPSM transcript. The Teg41 and αPSM promoters are found in close proximity to each other; therefore, it is tempting to speculate that AgrA and/or MgrA (which both bind in this region) may also have direct roles in activating the Teg41 promoter. We are currently investigating this possibility, mapping the Teg41 promoter and examining the expression of Teg41 under a variety of conditions. Previous work by Queck et al. (5) has shown that additional regulatory site(s) are located upstream of the AgrA binding site, within the Teg41 gene. We are also investigating the roles of these elements in the regulation of Teg41 in an effort to better understand how Teg41 controls αPSM production.

The data presented in this study clearly demonstrate regulation of the αPSMs by Teg41; however, additional studies will be required to fully understand the role of Teg41 in the S. aureus cell and to determine whether the regulation of Teg41 is transcriptional or posttranscriptional. We consider it likely that our inability to create a Teg41 deletion strain is due to the removal of critical promoter elements for the downstream *ndhF* gene while attempting to delete Teg41. Although not considered essential, it is likely that a strain deficient in *ndhF* expression would exhibit severe growth defects. Deleting 24 nucleotides at the 3′ end of Teg41 was possible, and the resulting strain (Teg41Δ3’) did not exhibit any growth defect compared to the wild type (see Fig. S3 in the supplemental material). This result strongly suggests that the *ndhF* promoter is intact in this strain and *ndhF* expression is unaffected. Even if *ndhF* expression is altered in the Teg41Δ3′ strain, our data demonstrate that it is not responsible for the hemolysis phenotype observed. Complementation of the Teg41Δ3′ hemolysis defect was possible with plasmid expression of Teg41 or Teg41_3′, demonstrating that the hemolysis phenotype is Teg41 dependent.

The work presented herein exemplifies the growing appreciation of the importance of sRNAs in S. aureus and further expands the list of sRNAs known to play a role in controlling virulence (10). It is the first time an sRNA has been shown to regulate expression of the αPSMs. Furthermore, it highlights the value of including annotations for sRNAs directly on reference genomes (11). Work is ongoing in our lab to fully explore the molecular mechanism through which Teg41 controls αPSM production. We acknowledge that a direct interaction between Teg41 and the αPSM transcript has yet to be established. We consider it likely that such an interaction exists; however, we do not discount the possibility that the regulation could be indirect. If this is the case, then the indirect regulation would appear to be mediated by the 3′ end of Teg41, as removal of this region resulted in a significant reduction in αPSM production, and expressing this region on a plasmid restores hemolysis in the Teg41Δ3′ strain. Regulation of αPSM production by Teg41 could potentially represent a novel target for therapeutic intervention, as disrupting Teg41 activity is likely to dramatically reduce the virulence potential of S. aureus.

## MATERIALS AND METHODS

### Strains, plasmids, and primers.

All bacterial strains and plasmids used in this study are listed in Table 1. Oligonucleotides are listed in Table 2. S. aureus cultures were routinely grown at 37°C with shaking in tryptic soy broth (TSB), and Escherichia coli cultures were grown at 37°C with shaking in lysogeny broth (LB). Staphylococcus epidermidis cultures were grown at 37°C with shaking in B2 broth (27). Where appropriate, the following antibiotics were used at the concentrations indicated: chloramphenicol (10 μg ml^−1^), erythromycin (5 μg ml^−1^), lincomycin (25 μg ml^−1^), and ampicillin (100 μg ml^−1^). To induce promoter activity in strains containing plasmid pCN51, CdCl_2_ was used at a concentration of 2 µM. For comparative analysis of supernatants, S. aureus cultures were synchronized as previously described (28). Briefly, overnight 5-ml cultures of each strain were diluted 1:100 in 10 ml of fresh, prewarmed TSB, and grown for 3 h to mid-exponential phase. The 3-h, mid-exponential-phase cultures were subsequently diluted into 25 ml of fresh TSB at a starting OD_600_ of 0.05. The cultures were then grown for the time indicated, typically 15 h.

**TABLE 1:** Bacterial strains and plasmids used in this study

Strain or plasmid	Relevant characteristic	Reference or source
*S. aureus* strains		
RN4220	Restriction-deficient transformation recipient	36
TCH1516	Community-associated USA300 MRSA isolate	37
UAMS-1	Osteomyelitis clinical isolate	38
SH1000	*rsbU* repaired laboratory strain	39
Newman	Laboratory strain	40
AH1263	USA300 LAC isolate cured of plasmid LAC-p03	41
JE2	USA300 LAC isolate cured of plasmids LAC-p01 and LAC-p03	42
RKC0602	UAMS-1 pMK4	This work
RKC0603	UAMS-1 pMK4_Teg41	This work
RKC0600	SH1000 pMK4	This work
RKC0601	SH1000 pMK4_Teg41	This work
RKC0535	AH1263 pMK4_Teg41	This work
RKC0604	Newman pMK4	This work
RKC0605	Newman pMK4_Teg41	This work
RKC0072	TCH1516 pMK4	This work
RKC0474	TCH1516 pMK4_Teg41	This work
RKC0494	JE2 pMK4	This work
RKC0470	JE2 pMK4_Teg41	This work
RKC0599	AH1263 pMK4	This work
RKC0538	AH1263 pMK4_Teg41	This work
RKC0630	AH1263 pCN51	This work
RKC0614	AH1263 pCN51_Teg41	This work
RKC0628	AH1263 pCN51_Teg41_3′	This work
RKC0552	AH1263 pMK4_Teg41_5′	This work
NE1354	USA300 JE2 *hla*::Bursa, *hla* NARSA transposon mutant	42
RKC0183	TCH1516 *hla*::Bursa, *hla* mutant	This work
RKC0472	TCH1516 *hla*::Bursa pMK4_Teg41	This work
RKC0521	AH1263 *hla*::Bursa, *hla* mutant	This work
BB2373	JE2 Δ*psmα*	B. Boles
RKC0504	JE2 Δ*psmα* pMK4	This work
RKC0442	JE2 Δ*psmα* pMK4_Teg41	This work
JLB162	AH1263 Teg41 3′ deletion (Teg41Δ3′)	This work
RKC0535	JLB162 pMK4_Teg41	This work
RKC0553	JLB162 pMK4_Teg41_5′	This work
RKC0631	JLB162 pCN51	This work
RKC0615	JLB162 pCN51_Teg41	This work
RKC0629	JLB162 pCN51_Teg41_3′	This work
*S. epidermidis* strains		
1457	Wild-type S. epidermidis	43
RKC0518	1457 pMK4	This work
RKC0519	1457 pMK4_Teg41	This work
*E*. *coli* strain		
DH5α	Cloning strain	Invitrogen

Plasmids		
pMK4	Gram-positive shuttle vector (Cm^r^)	44
pRKC0486	pMK4_Teg41 (vector overexpressing Teg41 from its native promoter)	This work
pRKC0554	pMK4_Teg41_5′ (vector overexpressing the 5′end of Teg41 from its native promoter, i.e., Teg41 missing the 3′ end)	This work
pCN51	Cadmium-inducible expression vector	45
pRKC0473	pCN51_Teg41 (vector overexpressing Teg41 from an inducible promoter)	This work
pRKC0628	pCN51_Teg41_3′(vector overexpressing the 3′ end of Teg41 from an inducible promoter)	This work
pJB38	Temperature-sensitive allelic exchange plasmid (Cm^r^)	46
pJB1037	pJB38 containing fragment upstream of Teg41	This work
pJB1039	pJB1037 containing fragment downstream of Teg41	This work

**TABLE 2:** Oligonucleotide primers

Primer	Sequence
JBKU89	CAGAATTCCACTCGCCAGTCGCAATATAAATAG
JBKU90	CAGGTACCATTATGTACAGAATCTACTATTGTAGG
JBKU92	CAGGTACCAGTTTAACTAGACACTGCATCACGGTAC
JBKU93	CAAGTCGACTTAAATTATTTTGCGAAAATGTCGATAATTGC
JBKU94	GTATCATGACAGCTAATACAAGTAGTACATTCGTC
JBKU95	CATCAATAAATCAACACAAAGCAAAGCCACCATC
#0232	CCGGAATTCAGATTACCTCCTTTGCTTATGAG
#0233	CGCGGATCCCCTACAATAGTAGATTCTGTAC
#0587	GGGAGCACGCTTAAAGAGAA
#0588	CAGAAAATTTACTATTGCAGGTTTCA
#0331	AGACACTGCATCACGGTACG
#0332	GGAATTCTTAAGCGTGCTCCCATGC
#0333	GGTCTCGTCTAGGCAAAGCA
#0334	GGAATTCGGGAGCACGCTTAAAGAGAA
#0301	ACGCGTCGACTTATGTTATAAGTTTAAAACG
#0302	GGAATTCGCAGAAAATTTACTATTGC
#0580	ACGCGTCGACGTGTCTAGTTAAACTGAAACCT
#0581	GGAATTCTAGTAGATTCTGTACATAATGGCA
#0488	CGCGGATCCCAGTTTAACTAGACACTGCATC
#0566	ACGCGTCGACTCACCTCACATCAATAAATCAACA
#0567	CGCGGATCCAAGCAAAGGAGGTAATCTTAATGG

### Plasmid and strain construction.

The Teg41-overproducing plasmid pMK4_Teg41 (pRKC0486) was constructed as follows. A 460-nt region of the S. aureus AH1263 chromosome (from 477,064 to 477,524), containing Teg41 and its native promoter, was amplified using primer pair #0232/#0233 and digested with the restriction enzymes EcoRI and BamHI. The resulting fragment was cloned into plasmid pMK4. The Teg41 5′ plasmid pMK4_Teg41_5′ (pRKC0554) was constructed as follows. A 408-nt region of the S. aureus chromosome, containing Teg41 and its native promoter but excluding 47 nt on the 3′ end, was amplified using primer pair #0232/#0488 and digested with the restriction enzymes EcoRI and BamHI. The resulting fragment was cloned into plasmid pMK4. The Teg41-overproducing plasmid pCN51_Teg41 (pRKC0473) was constructed as follows. A 205-nt region of the S. aureus chromosome, containing Teg41, was amplified using primer pair #0301/#0302 and digested with the restriction enzymes EcoRI and SalI. The resulting fragment was cloned into plasmid pCN51. The Teg41 3′ plasmid pCN51_Teg41_3′ (pRKC0628) was constructed as follows. A 62-nt region of the S. aureus chromosome, containing the 3′ end of Teg41, was amplified using primer pair #0580/#0581 and digested with the restriction enzymes EcoRI and SalI. The resulting fragment was cloned into plasmid pCN51.

After plasmid construction, clones were selected by transforming ligation mixtures into E. coli DH5α and selecting on ampicillin agar plates. Plasmid sequence was confirmed by restriction digestion and DNA sequencing. Once confirmed, plasmids were introduced into E. coli RN4220 by electroporation and subsequently transferred to additional strains by phage transduction (29). To introduce plasmids into S. epidermidis strains, total DNA (which contains both genomic DNA and plasmid DNA) was prepared from S. aureus strains RKC0072 and RKC0474 (30). Aliquots of each DNA isolation (which contain the desired plasmids) were then electroporated into S. epidermidis strain 1457 as previously described (31).

To construct the Teg41Δ3′ strain (JLB162), a chromosomal fragment upstream of Teg41 was amplified using PCR from the AH1263 chromosome with primers JBKU89 and JBKU90. The product was digested with EcoRI and KpnI and then ligated into the same site of pJB38 to generate pJB1037. Next, the downstream fragment was amplified using primers JBKU92 and JBKU93, digested with KpnI and SalI, and ligated into the same sites of pJB1037, yielding pJB1039. pJB1039 was introduced into strain RN4220 by electroporation and subsequently transferred into strain AH1263 by phage transduction as previously described (29). Allelic exchange was performed (32), and the mutation was confirmed by digestion with KpnI of a PCR product using primers flanking the mutation. Additionally, total DNA was extracted (30), and PCR was performed with primers JBKU94 and JBKU95 to amplify a region outside the sequence used for pJB1039. This region was completely sequenced to ensure that no unintended changes were made during the mutant construction process.

### Cell-free blood hemolysis assay.

Synchronized, cell-free, S. aureus culture supernatants were diluted 1:2 in reaction buffer (40 mM CaCl_2_, 1.7% NaCl) and incubated at 37°C in a tube revolver with 25 μl of whole blood from humans or rabbits. Following a 10-min incubation, the samples were centrifuged at 5,500 × *g*, and 100 μl of the supernatant was transferred to a 96-well plate. The degree of erythrocyte lysis was determined by reading the absorbance of the samples at OD_543_.

### Whole-blood hemolysis assay.

Bacterial strains were grown to mid-exponential phase, pelleted, washed with PBS, resuspended in PBS, and used to inoculate 2-ml aliquots of human blood at an equivalent of OD_600_ of 0.05. Inoculated samples were incubated with agitation at 37°C. At the time points indicated, 200-μl samples were withdrawn from each sample, and the intact erythrocytes were pelleted by centrifugation at 10,000 rpm. The degree of erythrocyte lysis was determined by OD_543_ measurement of the resulting supernatant. At each time point, additional samples were withdrawn, and the number of bacteria was determined by serially diluting and plating on TSA.

### Butanol extraction of PSMs.

PSMs were isolated from S. aureus culture supernatants using the previously published butanol extraction method (18). Five milliliters of synchronized cell-free culture supernatants was combined with 2 ml of *n*-butanol. The samples were incubated with shaking at 37°C for 1 h. After this incubation, the top organic layer was removed and dried down via vacuum centrifugation for 6 h at 5,000 rpm. To visualize PSMs, extracts were dissolved in water, mixed with 6× loading buffer, separated on 12% SDS-PAGE gels, and stained with Coomassie brilliant blue.

### Whole-genome sequencing.

To confirm that the 24-nucleotide region of Teg41 was successfully deleted and to ensure that no additional inadvertent deletions had occurred, we had the genome of strain JLB162 and the parent AH1263 strain prepared and sequenced on an Illumina MiSeq at the Ohio University Genomics Facility. DNA quantity was determined using the Qubit HS DNA kit (catalog no. Q32854; ThermoFisher) and the Qubit 3.0 fluorometer (ThermoFisher). Genomic DNA was diluted to 0.2 ng/µl, and paired-end libraries were prepared using the Illumina Nextera XT DNA library prep kit (catalog no. FC-131-1024; Illumina) according to the manufacturer’s instructions. Library quality was assessed on the Agilent 2100 bioanalyzer (Agilent) using a High Sensitivity DNA chip (catalog no. 5067-4626; Agilent). As all libraries were of high quality and concentrations were >15 ng/µl, samples were normalized and pooled using the bead-based method according to the Illumina protocol (document 15031942 v02, Illumina). Libraries were sequenced on the Illumina MiSeq (Illumina) using the MiSeq reagent kit v2 (500 cycles) 2 × 250-bp paired-end sequencing (catalog no. MS-102-2003; Illumina). FastQ files were generated and retrieved from BaseSpace Sequencing Hub.

### Bioinformatics.

Analysis of whole-genome sequencing data was performed using CLC Genomics Workbench. Sequencing reads for the wild-type AH1263 (parent strain) and the Teg41Δ3′ mutant were aligned to the USA300_FRP3757 genome, and variants were detected using the “Basic variant detection” function. Variants that were common to both strains were eliminated, generating a list of variants specific to the Teg41Δ3′ mutant.

### Mouse skin and soft tissue infection.

A subcutaneous infection model was performed as previously described (33). BALB/c mice were anesthetized by isoflurane inhalation on the day of infection, shaved, and treated with Nair on the right flank to remove fur. Mice were injected subcutaneously with 50 μl of saline solution containing 6.5 × 10^7^ CFU. After 7 days, mice were sacrificed by CO_2_ asphyxiation, and abscesses were removed. Excised abscesses were weighed to the nearest 0.001 g and measured to the nearest 0.01 mm with a digital caliper and homogenized. Homogenates were serially diluted and plated to determine the recovered number of CFU/gram in abscesses.

### Histopathology.

Murine skin abscesses were excised and fixed in 10% neutral buffered formalin. Excised tissue was embedded in paraffin, sectioned on a microtome, and stained with hematoxylin and eosin. All pictures were taken at a magnification of ×20 for analysis.

### Mass spectrometry proteomics.

Dry samples of PSMs were dissolved in water and diluted with 100% methanol and 0.5% formic acid at a ratio of 1:4 sample-methanol. Direct infusion mass spectrometry was carried out in an automated fashion at a flow rate of 5 μl/min using a Q-Exactive HF mass spectrometer (Thermo Fisher) fitted with a heated electrospray source (HESI) and running a 2-min Top20 method with 10-s dynamic exclusion at a resolution of 120,000 and 30,000 for MS1 and MS2, respectively. The maximum MS2 ion time was set at 200 ms with an AGC of 1e^6^ and an isolation window of 1.2 *m/z*.

Spectra were extracted from raw data files and converted into Mascot Generic Format (mgf) files using Proteome Discoverer 2.2. (Thermo Fisher) and then analyzed using the search engine Mascot (version 2.6.1; Matrix Science, London, UK). Mascot was set up to search a database containing the PSM sequences and the Uniprot_S_aureus_USA300 database by setting the digestion enzyme as “nonspecific” and using a fragment ion mass tolerance of 0.060 Da and a parent ion tolerance of 10 ppm. Oxidation of histidine, methionine, and tryptophan, cations Na and K of aspartic acid and glutamic acid, and formylation of the N terminus were specified in Mascot as variable modifications. The total ion current for each peptide and charge state were also determined using XCalibur 4.1.

### Northern blotting.

RNA isolation was performed at the time points indicated as previously described by our group (34). For Teg41 Northern blots, 10 μg RNA was separated on a 10% polyacrylamide gel and transferred to a nylon membrane by electroblotting as previously described (11). For αPSM Northern blots, 10 μg RNA was separated on a 1% agarose gel containing 1× MOPS and 6.6% formaldehyde. RNA was transferred to a nylon membrane by capillary transfer using 20× SSC buffer and immobilized by UV irradiation. Radiolabeled probes for Teg41 and the αPSM transcript were generated using the Roche random prime labeling kit (Sigma). Teg41 was amplified by PCR using primers #301 and #302, and the αPSM transcript was amplified using primers #566 and #567. Approximately 1 μg of purified PCR product was used in each labeling reaction with [α-^32^P]ATP according to the manufacturer’s protocol. Unincorporated radiolabeled nucleotide was removed, and probes were purified using Illustra MicroSpin G-25 columns (GE Healthcare). Membranes were prehybridized for 1 h at 45 °C in ULTRAhyb-Oligo buffer and then incubated with radiolabeled probe overnight at 45°C. After incubation, membranes were washed with 2×, 1×, and 0.5× SSC buffer and visualized using a phosphorimager screen.

### Reverse transcriptase-quantitative PCR (RT-qPCR).

Three biological replicates of strain AH1263 and the Teg41Δ3′ strain were grown, and total RNA was isolated at 3 h and 6 h after subculture. Complementary DNA (cDNA) was generated from 1 μg of total RNA using iScript reverse transcriptase (Bio-Rad) according to the manufacturer’s instructions. The cDNA was diluted 10 times and used in SYBR Green reactions (Bio-Rad) in technical duplicates to analyze the expression of Teg41 and the αPSM transcript according to the manufacturer’s instructions. Amplification and analysis were performed as described previously (35). The housekeeping gene *gyrB* was used as the endogenous control in all RT-qPCR reactions.

### Ethics statement.

Human blood samples were obtained from donors at Ohio University. All collections, handling, and usage of blood was approved by the Ohio University Institutional Review Board. Whole rabbit blood was purchased from Hemostat Laboratories. Six-week-old BALB/c mice were ordered from Envigo and held at the Ohio University Office of Laboratory Animal Resources. All animal work was done by trained lab personnel and approved by the Institutional Animal Care and Use Committee.

### Data availability.

Whole-genome sequencing read data have been deposited in the SRA database (SubmissionID SUB3843925; BioProject ID PRJNA445980).
