# Supplementary figures and images for: The Small RNA Teg41 Regulates Expression of the Alpha Phenol-Soluble Modulins and Is Required for Virulence in Staphylococcus aureus
Source: mBio. 2019 Feb 5;10(1):e02484-18. doi: 10.1128/mBio.02484-18 (PMC6428751; doi:10.1128/mBio.02484-18)

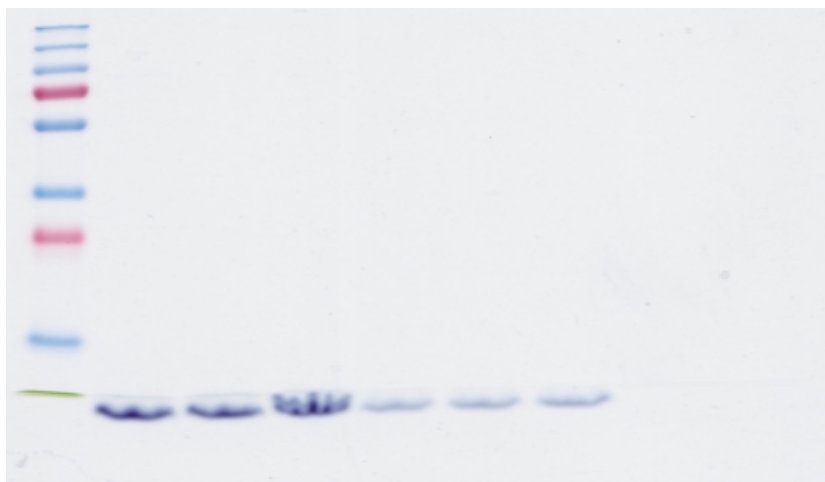

**Figure S1.** Full length gel from Figure 5B.

Supplement: FIG S1 [file mBio.02484-18-sf001.pdf]
