# Supplementary material for: The Small RNA Teg41 Regulates Expression of the Alpha Phenol-Soluble Modulins and Is Required for Virulence in Staphylococcus aureus
Source: mBio. 2019 Feb 5;10(1):e02484-18. doi: 10.1128/mBio.02484-18 (PMC6428751; doi:10.1128/mBio.02484-18)
Supplement: FIG S2 [file mBio.02484-18-sf002.pdf]

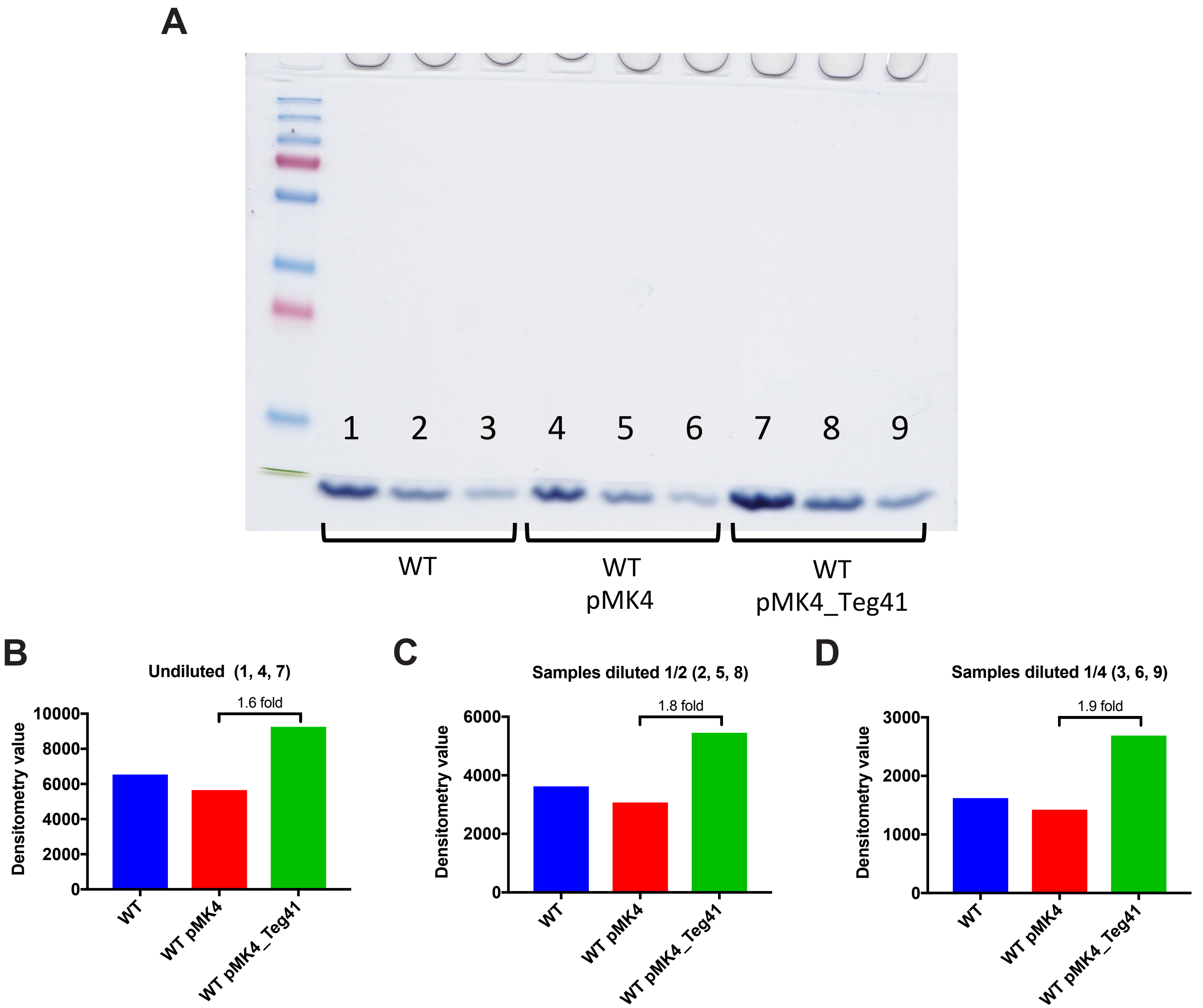

Figure S2. Densitometry analysis of butanol extracted PSMs. **A.** SDS-PAGE of butanol extracted PSMs from wild type *S. aureus* (WT), vector control (WT pMK4), and Teg41 overproducing strains (WT pMK4_Teg41). Lanes 1, 4, and 7 are undiluted samples. Lanes 2, 5, and 8 are samples diluted ½. Lanes 3, 6, and 9 are samples diluted ¼. **B.** Densitometry analysis of lanes 1, 4, and 7. **C.** Densitometry analysis of lanes 2, 5, and 8. **D.** Densitometry analysis of lanes 3, 6, and 9.
