# Supplementary material for: The Small RNA Teg41 Regulates Expression of the Alpha Phenol-Soluble Modulins and Is Required for Virulence in Staphylococcus aureus
Source: mBio. 2019 Feb 5;10(1):e02484-18. doi: 10.1128/mBio.02484-18 (PMC6428751; doi:10.1128/mBio.02484-18)
Supplement: FIG S3 [file mBio.02484-18-sf003.pdf]

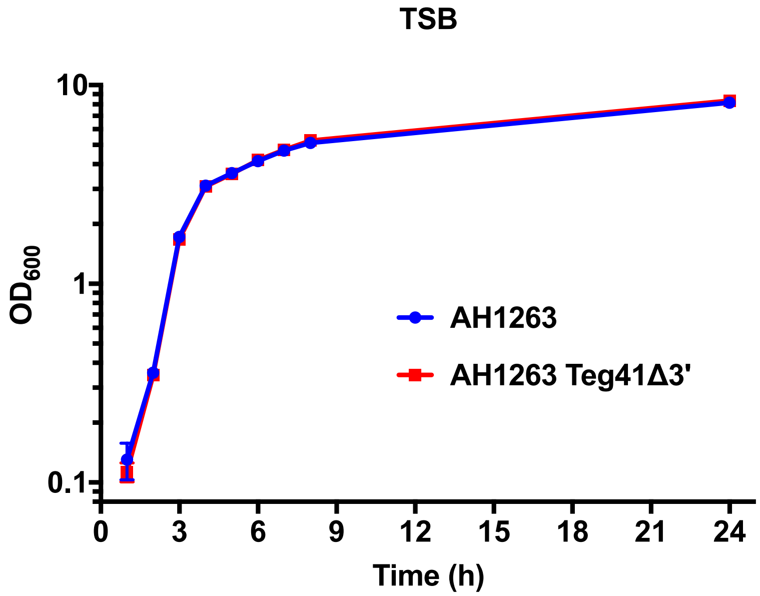


**Figure S3.** Growth of wild type *S. aureus* and the Teg41Δ3' mutant in TSB. No significant difference in growth was observed in either condition.
